# Cellulose-Reinforced Polylactic Acid Composites for Three-Dimensional Printing Using Polyethylene Glycol as an Additive: A Comprehensive Review

**DOI:** 10.3390/polym15193960

**Published:** 2023-09-30

**Authors:** Kelly Cristina Coelho de Carvalho Benini, Anne Shayene Campos de Bomfim, Herman Jacobus Cornelis Voorwald

**Affiliations:** Fatigue and Aeronautical Materials Research Group, Department of Materials and Technology, UNESP-São Paulo State University, Guaratinguetá, São Paulo 12516-410, Brazil; anne.shayene@unesp.br (A.S.C.d.B.); h.voorwald@unesp.br (H.J.C.V.)

**Keywords:** polylactic acid, polyethylene glycol, cellulose, 3D printing

## Abstract

Growing concerns about environmental issues and global warming have garnered increased attention in recent decades. Consequently, the use of materials sourced from renewable and biodegradable origins, produced sustainably, has piqued the interest of scientific researchers. Biodegradable and naturally derived polymers, such as cellulose and polylactic acid (PLA), have consistently been the focus of scientific investigation. The objective is to develop novel materials that could potentially replace conventional petroleum-based polymers, offering specific properties tailored for diverse applications while upholding principles of sustainability and technology as well as economic viability. Against this backdrop, the aim of this review is to provide a comprehensive overview of recent advancements in research concerning the use of polylactic acid (PLA) and the incorporation of cellulose as a reinforcing agent within this polymeric matrix, alongside the application of 3D printing technology. Additionally, a pivotal additive in the combination of PLA and cellulose, polyethylene glycol (PEG), is explored. A systematic review of the existing literature related to the combination of these materials (PLA, cellulose, and PEG) and 3D printing was conducted using the Web of Science and Scopus databases. The outcomes of this search are presented through a comparative analysis of diverse studies, encompassing aspects such as the scale and cellulose amount added into the PLA matrix, modifications applied to cellulose surfaces, the incorporation of additives or compatibilizing agents, variations in molecular weight and in the quantity of PEG introduced into the PLA/cellulose (nano)composites, and the resulting impact of these variables on the properties of these materials.

## 1. Introduction

Polylactide, also known as poly(lactic acid) (PLA), stands as a biopolymer derived from renewable and biodegradable sources. It boasts remarkable mechanical characteristics, including a notable ultimate tensile strength ranging from 50 to 70 MPa and a tensile modulus of around 3 GPa [1,2,3]. These attributes outshine those of various polymers sourced from petroleum. However, PLA does exhibit certain drawbacks. These encompass inherent low strength and toughness, limited thermal resistance denoted by a thermal deflection temperature of 55 °C, as well as susceptibility to melting and a slow crystallization process. Consequently, these limitations restrict its application in fields like automotive and electronics [1,2,3,4].

To overcome these disadvantages, recent research has investigated the use of cellulose to improve the mechanical, thermal, and rheological properties of PLA [1,4,5,6,7,8,9,10]. Despite the promising results, many studies are still needed to address the problems of lack of compatibility with hydrophilic cellulose and hydrophobic polymeric matrices, agglomeration problems that cellulose presents during the drying of aqueous suspensions and dispersion in the melted polymeric matrix. Additionally, the impact of cellulose filler on melt flow within the polymeric matrix, potentially disrupting processes like injection molding, compression molding, and 3D printing, necessitates attention [1,4,5,6].

The use of cellulose as a natural reinforcement for PLA has been widely studied, as it provides the PLA matrix with better mechanical and rheological properties as well as high crystallinity, which depends on the combination of different factors, such as the mixing processes used and the properties of the cellulose (morphology, size, isolation methods, drying, and surface modifications), and the use of compatibilizing agents, surfactants and plasticizers [1,5,7]. Combining these different factors allows the acquisition of materials with specific properties and guarantees a better distribution of cellulose in the polymeric matrix and better adhesion between cellulose and PLA. A good dispersion of these materials in the PLA matrix, for example, can favor the transfer of stress from the matrix to the cellulose and improve the mechanical properties, as well as a change in the mobility of the chains and an increase in the rate of crystallization and crystallinity, provided by adding reinforcement, can affect the thermomechanical and barrier properties [8]. 

When producing PLA composites with cellulose, it is common to incorporate additives that enhance material compatibility and modify melt viscosity. This is particularly important as introducing reinforcement, depending on its scale and quantity, has the potential to elevate viscosity, thereby potentially impeding the efficiency of manufacturing processes. One of the most used additives for this type of composite is polyethylene glycol (PEG), which, depending on the molecular weight, can act both as a compatibilizing agent and as a plasticizer [9]. In addition to PEG, other additives can be used to improve the flexibility and impact resistance of PLA, such as citrate esters, glycerol, epoxide palm oils, poly(propylene glycol), oligomeric acid lactic [10], oligomeric malonate ester amides, and glucose monoesters [11,12].

Regarding the methods used in the preparation of PLA/cellulose composites and nanocomposites, the most common are melt mixing (internal mixer, single or twin-screw extruder) [13,14,15,16], solvent evaporation by casting [5,17], or a combination of two or more processes. Among these, melt mixing using an internal mixer or twin-screw extruder has the most potential for industrial application since solvent evaporation by casting is used only on a laboratory scale [1]. After mixing and attainment of the composite, various molding techniques can be applied, including electrospinning [9], injection molding [18], compression [19], and, more recently, additive manufacturing methods [16,20,21,22,23,24,25].

Additive manufacturing (AM) techniques, which include fused filament fabrication (FFF), are gaining prominence in recent years because they have advantages such as precise control of complex structures, the ability to use various types of plastic materials, and little waste in the process [24].

Hence, this review provides a comprehensive analysis of the existing literature pertaining to the utilization of cellulose in various scales, concentrations, and forms to reinforce the biodegradable PLA matrix. It also explored the impact of incorporating PEG as an additive and examined the application of these composite materials in filament production for 3D printing. Furthermore, this review systematically addresses the array of studies within the literature encompassing the following themes: cellulose-reinforced PLA composites, the role of PEG as an additive to enhance and modify composite properties, the expansion of processing possibilities and potential applications, and the utilization of PLA/cellulose/PEG (nano)composites for filament fabrication in the realm of 3D printing.

## 2. Materials and Methods

Biopolymers are defined as sustainable polymers produced from a variety of renewable raw materials such as polysaccharides, lignin, vegetable oils, pine resin derivatives, and proteins as a substitute for petroleum, the most conventional fossil resource [26,27]. This class of biomaterials includes biofibers, biopolymers, and biocomposites [28], and the term “bio” is used to designate a biodegradable material and materials from renewable sources. Biopolymers can be divided into biodegradable, which are not bio-based (synthetic polyesters and polyvinyl alcohol (PVA)), biodegradable and bio-based (starch, cellulose, PLA, polyhydroxyalkanoate (PHA), and poly(3-hydroxybutyric acid) (PHB)), and bio-based but not biodegradable (biobased polydioxanone (PDO), glycerol, polyethylene (PE), polyethylene 2,5-furandicarboxylate (PEF), polyvinyl chloride (PVC) from bioethanol, derived polycarbonates (PCs), and polyamides (PAs) from oils) [27,29,30].

The biopolymer market has experienced growth in recent years, encompassing both biodegradable and non-biodegradable variants derived from renewable and non-renewable sources. This expansion is attributed to the increasing demand from consumer goods companies, who are striving to offer environmentally friendly products in alignment with sustainability standards [31,32].

According to the European Bioplastic Report [33], the total production volume of bio-based polymers, biodegradable or not, in 2019 was approximately 2.1 million tons and could reach 2.4 million in 2024. Concerning the global bioplastics production capacity, 45% is in Asia and 12% in South America.

Despite the promising growth, the production of bioplastics still represents less than 1% of the more than 390 million tons of plastic produced annually. This limitation persists due to factors such as the elevated production expenses, insufficient political incentives, and inherent properties that frequently hinder their compatibility with certain applications across various sectors [31,32]. 

Among the currently most used biopolymers, PLA stands out for being obtained from a renewable source and presenting biodegradability in industrial composting environments [6]. PLA is classified as an aliphatic thermoplastic polyester, synthesized from monomers derived through carbohydrate fermentation of renewable resources like corn, cassava, starch, sugar cane, and sugar beet [34,35]. 

Due to the chirality of the lactic acid monomer, PLA exists in different enantiomeric forms (L-PLA, D-PLA, and LD-PLA), and its properties depend both on the content of optical impurities among the enantiomers and the molecular weight [36]. Polymers with more than 88% enantiomerically pure monomers are semicrystalline and have better thermomechanical properties [37]. Commercially available PLA is usually produced from L-LA monomers, and the presence of D-LA monomers in the chain acts as a defect and tends to decrease the crystallinity and melting temperature of the polymer [1,8].

The main applications of PLA are in the production of plastic films, recyclable and biodegradable packaging (bottles, yogurt cups, and candy wrappers), garbage bags, wall coverings, fabrics, fibers, and biodegradable medical devices (e.g., sutures, catheters, and prosthetic materials), textile fibers, and 3D printing filaments [3,38]. 

Although it has lower toughness; less than 10% elongation compared to conventional polyolefins such as PP, for example; and a low crystallization rate, these properties can be improved with the addition of different filler particles, including cellulose at micro- and nanoscales, which, in addition to improving the thermomechanical properties, do not affect the biodegradability of the polymer [3,5,36,39,40,41]. 

Regarding its application as a filament in additive manufacturing, PLA offers several advantages outlined in Table 1. These include its ease of printing, exceptional visual quality, minimal warping issues, notable tensile strength, and a comparatively lower coefficient of thermal expansion when compared to alternative polymers like ABS, polyethylene terephthalate glycol (PETG), and PP.

Nonetheless, certain limitations, also listed in Table 1, hinder PLA’s widespread applicability. Notably, it presents drawbacks like higher costs, elevated hardness and rigidity, constrained service temperature range, diminished toughness, limited crystallization capacity, increased porosity, and insufficient inter-layer adhesion. These factors collectively curtail its potential usage in specific applications [3,15].

## 3. Cellulose as a Reinforcement of PLA Matrix Composites

In polymer composites with a PLA matrix, the cellulose used as filler or reinforcement is added in different amounts, shapes, and scales, such as cellulose fibers [13,14,16,20,42,43,44,45], microcrystalline cellulose (MCC) [5,6,17,46,47,48], microfibrillated cellulose (MFC) [10,49,50], nanocrystalline cellulose (NCC) [24,51,52], and nanofibrillated cellulose (CNF) [25,53,54]. The scale of the cellulose, as well as the amount added in the polymeric matrix are responsible for the different final properties of the polymeric composites. According to Aumnate et al. [20], the smaller size of the cellulose can enhance the mechanical properties, and the larger size of the cellulose could provide good formability, network structure, and entities.

Cellulose can be derived from diverse natural sources like sugarcane bagasse [44,45], flax [14], bamboo [14], wood, sugar palm fiber [16], and ramie [43]. Typically, it is incorporated into the PLA matrix at concentrations varying between 1 and 30 wt.%, contingent on its morphology and dimensions [42]. The addition of natural cellulose within polymeric matrices holds significant appeal due to its capacity to enhance mechanical and thermal characteristics [55]. Furthermore, this approach offers distinct advantages, including low density, remarkable specific mechanical properties, minimal toxicity, biocompatibility, and biodegradability [24,56].

Cellulose fibers are defined as a set of microfibrils where the cellulose molecules are stabilized laterally through hydrogen bonds between the hydroxyl groups [57]. In the literature, the term microfibril, which refers to a crystalline cellulose bound by amorphous regions, despite the prefix micro, is usually used to describe cellulose fibers 2–10 nm in diameter and several tens of micrometers in length, depending on the source from which the fiber was extracted [58,59]. The terms nanofibril and nanofiber are also used as synonyms for microfibril. The term microfibrillated cellulose, with a diameter between 10 and 40 nm and a length greater than 1000 nm, is defined as an aggregate of cellulose microfibrils obtained by the disintegration of cellulose fibers [60,61]. The term whiskers or cellulose nanocrystals is used to define nanocrystalline cellulose in the form of rods with a diameter between 2 and 20 nm and a length between 100 and 600 nm [61].

The isolation of cellulose nanofibers from lignocellulosic fibers can be performed via different processes, such as electrospinning [62,63,64,65], mechanical processes [66], acid or enzymatic hydrolysis [65,67,68,69], as well as the combination of two or more processes [58].

Distinct morphologies are achieved contingent on the technique employed to fragment macroscopic fibers into nanofibers. Through acid hydrolysis, a colloidal suspension of aggregates characterized by high crystallinity and an elevated aspect ratio is formed, referred to as microcrystalline cellulose (MCC). Subsequent to acid hydrolysis, the application of sonification disintegrates the fibril aggregates, yielding cellulose whiskers, also recognized as nanocrystalline cellulose (NCC). Utilizing multiple mechanical shearing actions yields microfibrillated cellulose (MFC) or nanofibrillated cellulose (CNF), both comprising interconnected fibrils and fibril aggregates. Additionally, MFC can be obtained through pretreatment involving enzymatic hydrolysis coupled with mechanical shearing [65].

Regardless of the cellulose scale used, there is a chemical incompatibility between hydrophilic cellulose and most hydrophobic thermoplastic polymer matrices. Thus, to improve the compatibility between cellulose and the PLA matrix, surface treatments are often used, which can be chemical, physical, or enzymatic and whose primary function is to increase the hydrophobicity of the cellulose surface, removing some amorphous constituents from lignocellulosic sources or adding some functional group that increases the hydrophobicity of the fiber and improves the chemical and mechanical compatibility with the polymeric matrix. It is also possible to graft polymers on a cellulose surface, decreasing the surface energy and increasing compatibility between cellulose and a polymer matrix [70].

The use of nanocellulose as a natural reinforcement for PLA has been widely studied, as it provides the PLA matrix with better mechanical and rheological properties, which depend on the combination of different factors, such as the mixing processes used and the properties of the cellulose (methods of isolation, drying, and surface modifications), as well as the use of compatibilizing agents and surfactants [1,7]. Combining these distinct factors, the acquisition of materials endowed with precise properties is facilitated. This approach ensures an enhanced dispersion of cellulose within the polymeric matrix and fosters improved adhesion between cellulose and PLA.

## 4. Poly(ethylene glycol) (PEG) as a Plasticizer of PLA

The use of plasticizers is one of the effective methods to change the properties of polymers and thus increase the variety of applications. PLA is a rigid polymer, which limits its use in many applications, and in this way the use of plasticizers improves the mechanical properties and also helps in the fluidity of the polymeric matrix during processing [71]. Plasticizers reduce intermolecular forces, heighten the mobility of polymer chains, and consequently enhance thermomechanical properties [71].

Among the various plasticizers used for PLA, notable choices include citrate esters, poly(ethylene glycol) (PEG), glycerol, oligomeric lactic acid, and glucose monoesters. PEG, particularly when possessing low molecular weight, distinguishes itself due to its exceptional miscibility, biodegradability, and suitability for applications involving food contact [11,12].

PEG is a nonionic, water-soluble polymer, characterized by the general formula H(OCH_2_CH_2_)_n_OH. It is available in a broad range of molecular weights, spanning from 100 to 6000 g/mol [11,71]. In the literature, PEG with diverse molecular weights, encompassing the 100 to 6000 g/mol range, has been employed as a PLA plasticizer for crafting composites and nanocomposites infused with cellulose fillers. The elevation of molecular weight enhances resistance to additive migration towards the material’s surface, thereby extending its lifespan. Conversely, this elevation diminishes the likelihood of volatilization [72]. 

PEG as an additive for composites with a PLA matrix can be used both as a plasticizer, to improve the fluidity of the polymer, simplifying the mixing and molding in different manufacturing processes, and also as a compatibilizing agent, improving the adhesion between the cellulose and the PLA matrix [9].

Jacobsen et al. [73] studied the effect of different plasticizers on the mechanical properties of PLA. In addition to PEG1500, glucosemonoester and fatty acids were both used in proportions of 2.5, 5, and 10 wt.%. The authors reported that the elastic modulus and tensile strength of the material decreased with the increase in the percentage of PEG, while the strain at break increased, with the increase being greater for 10% of PEG. The impact strength also increased with the addition of 10% PEG, while the addition of PEG1500 decreased the value of glass transition temperature.

Chieng et al. [11] used PEG with low molecular weight (Mn = 200 g/mol) to plasticize PLA, maintaining the plasticizer content at the maximum 10 wt.%. According to the authors, low-molecular-weight PEG increases miscibility with the polymer and is more efficient in reducing the glass transition temperature. In this study, the addition of PEG considerably increased the elongation at break of PLA (>7000%) but decreased the tensile strength and tensile modulus. The use of low-molecular-weight PEG decreases the intermolecular force and increases the mobility of the polymeric chains, improving the flexibility and plastic deformation of PLA. According to the TGA results, the authors also observed that the addition of PEG decreased the thermal stability of PLA since the plasticizer intercalates with the polymer and breaks the polymer–polymer interactions.

Luangtana-Anan et al. [71] studied the effect of molecular weight and concentration of polyethylene glycol on physicochemical properties and stability of shellac film. Different molecular weights of PEG (200, 400, and 4000 g/mol) were chosen at a concentration of 10% *w*/*w*. PEG400, with its appropriate molecular weight, was found to effectively shield the carboxylic and hydroxyl groups of the shellac chain, demonstrating the significance of plasticizer molecular weight in protecting active sites. Examining different concentrations of PEG400, it was observed that a 10% (*w*/*w*) concentration could maintain stability for four months, after which considerable parameter shifts occurred. Notably, an increased concentration of PEG400, specifically 20% (*w*/*w*), extended shellac stability for 6 months.

Li et al., 2020 [74] studied the effect of molecular weight of PEG on crystallization behaviors and thermal properties of triblock copolymer polylactid acid stereocomplexes. The results confirmed the formation of polylactide stereocomplexes in the PLLA blends (PEG–PDLA/PLLA).

## 5. Additive Manufacturing and 3D Printing Technology

In Industry 4.0, 3D printing manufacturing technology, an additive manufacturing technique, also known as layered deposition or rapid prototyping, is a process for manufacturing three-dimensional (3D) objects through the deposition of material in layers, capable of producing parts with complex and customized ergonomic shapes [75,76]. Within 3D printing technologies, such as stereolithography, selective laser sintering, and fused filament manufacturing (FFF), the latter has stood out for personal, educational, and professional use in the manufacture of components using thermoplastic polymers or composites due to its characteristics such as low cost, ease of use, potential to reinvent the design process, high degree of automation, and reproducibility [76,77,78].

The thermoplastics most used in this type of additive manufacturing are ABS and PLA, and PLA is more sustainable since it is derived from natural sources and is biodegradable in an industrial composting environment. Furthermore, parts produced by 3D printing using the FFF technique with PLA have fewer warping problems and better mechanical properties compared to ABS [77]. Conversely, PLA exhibits certain drawbacks in 3D printing applications, including high porosity and inadequate inter-layer adhesion [77], limited thermal stability, reduced crystallization capability, drawability, poor toughness, and a deficiency in flexibility and elasticity [24]. Within this framework, the incorporation of cellulose reinforcements emerges as a feasible alternative, enhancing PLA’s attributes while upholding the polymer’s biodegradability and environmental nature [1]. However, a major disadvantage of using composites is the difficulty in obtaining homogeneous filaments in diameter and in relation to the reinforcement distribution, significantly affecting 3D printability of the composite filaments and the physical properties of the 3D-printed products [20].

Despite the numerous papers published in this area, much development still needs to be carried out since problems of cellulose agglomeration, the lack of compatibility with the polymeric matrix, and the heterogeneity of the cellulose dispersion within the matrix, as previously mentioned, minimize the capacity of the cellulose to improve the mechanical and rheological properties of PLA while also affecting the printability of the polymer, inducing processing problems, color change, and carbonization [52].

## 6. PLA/Cellulose (Nano)composites for Filament Production—Systematic Review Considering PEG as an Additive and 3D Printing

### 6.1. Methodology

#### 6.1.1. Purpose

The primary aim of this study is to explore and comprehend the pivotal role of PEG in enhancing the properties of PLA/cellulose (nano)composites when employed in the fabrication of filaments intended for 3D printing. A comprehensive analysis is sought on how the incorporation of PEG affects the mechanical, thermal, and rheological characteristics of these composites, as well as their overall performance as raw materials in additive manufacturing. To evaluate the state of the art in this subject, a bibliometric analysis was carried out of published articles that contained some specific keywords within this theme.

#### 6.1.2. Scope

A systematic literature search was conducted in the Scopus and Web of Science (WOS) databases, considering English-language documents, and excluding conference papers, reviews, and book chapters. Four distinct searches were carried out using the keywords outlined in Table 2, referred to as Searches 0, 1, 2, and 3. Table 2 provides details on the number of unique documents found, after excluding duplicates identified in both databases, along with their respective publication years.

#### 6.1.3. Function

The primary function of this systematic review article is to comprehensively investigate and analyze the effects of incorporating PEG into cellulose-reinforced PLA (nano)composites, particularly in the context of filament production for 3D printing. The review critically evaluates the existing literature to understand how different characteristics of PEG, such as molecular weight, percentage added, and the type of cellulose used, influence the mechanical, thermal, and rheological properties of these composites. Additionally, it seeks to identify trends and usage patterns of PEG in these composites by synthesizing findings from a range of studies. The review also highlights research gaps, indicating areas that require further investigation.

Furthermore, the article serves as a decision-support tool for optimizing PLA/cellulose composite formulations for 3D printing by providing valuable insights based on a systematic analysis of the literature.

#### 6.1.4. Intent

This review is intended to provide an informative synthesis of the existing literature, aiming to offer insights and understanding rather than making prescriptive recommendations. Its primary purpose is to comprehensively analyze and distill the available knowledge, facilitating a deeper understanding of PLA/cellulose composites with PEG in 3D printing, without prescribing specific actions or outcomes.

### 6.2. Results

#### 6.2.1. Bibliometric Analysis

The initial search (Search 0) considered general terms, resulting in 1346 documents spanning the past 45 years. Subsequently, the inclusion of the term “PEG” (Search 1) notably reduced the pool to 51 documents published within the last seventeen years. With the incorporation of the term “3D printing” (Search 2), the search yielded 45 documents, all of which are notably recent, originating from 2017 onwards. Finally, the most recent search (Search 3) combined “PEG” and “3D printing”, culminating in the discovery of only two documents published in the last seven years. This clearly underscores a research gap in the literature, presenting a significant opportunity for future research.

**Figure 1:**
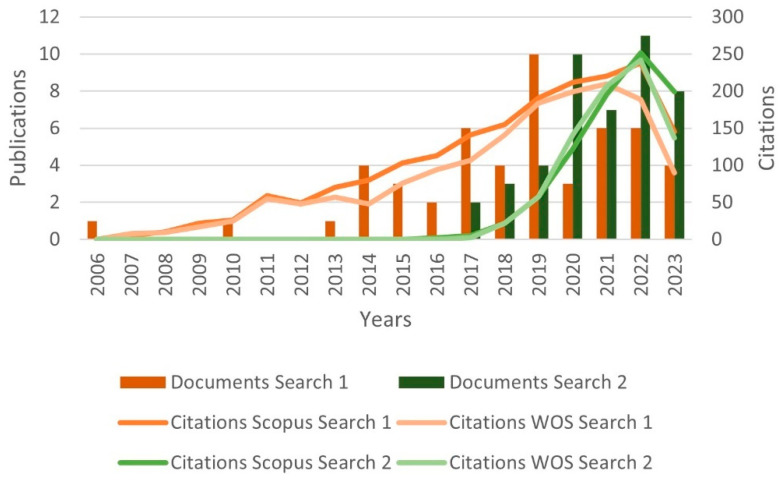
Number of publications and citations over the years, according to Scopus and Web of Science databases.

Regarding Searches 1 and 2, Figure 1 illustrates the cumulative documents and citations over the years. In Search 1, the number of documents began to rise from 2013 and peaked in 2019 with 10 publications. Subsequently, a declining trend has been observed up to the present. This pattern underscores the subject’s sustained presence in the literature for over a decade, underscoring its enduring scientific significance. On the other hand, Search 2 addresses a contemporary topic, commencing from 2017, demonstrating a pronounced upsurge in document count, particularly since 2020. This trajectory underscores the scientific significance of both cellulose-reinforced PLA composites and the evolving trend of 3D printing. The citation curves accentuate the ongoing scientific importance of these subjects in the international literature, indicating a consistent upward trajectory over the years.

Furthermore, Table 3 emphasizes the five most cited documents from Searches 1 and 2. Notably, Search 1 documents have a higher number of citations, some of which have been cited since 2007. The first document was published in 2006 and has earned attention and relevance over the years with 680 citations. Nonetheless, documents of Search 2, being a more recent area of focus (beginning in 2017), have shown a notable rise in citations in the last three years, with document citations ranging between 60 and 140. The document with the highest citation count was published in 2018, with 143 citations. Additionally, a significant observation is that most documents from both searches originate from Asia (six) and Europe (three), highlighting the scientific prominence of research groups from these continents. Notably, one document from Search 3 is among the five most cited documents from Searches 1 and 2. This document, titled “Preparation of 3D printable micro/nanocellulose-polylactic acid (MNC/PLA) composite wire rods with high MNC constitution” [17], underscores the growing representation and increasing significance of the topic of cellulose-reinforced PLA composites incorporating PEG for 3D printing applications.

**Figure 2:**
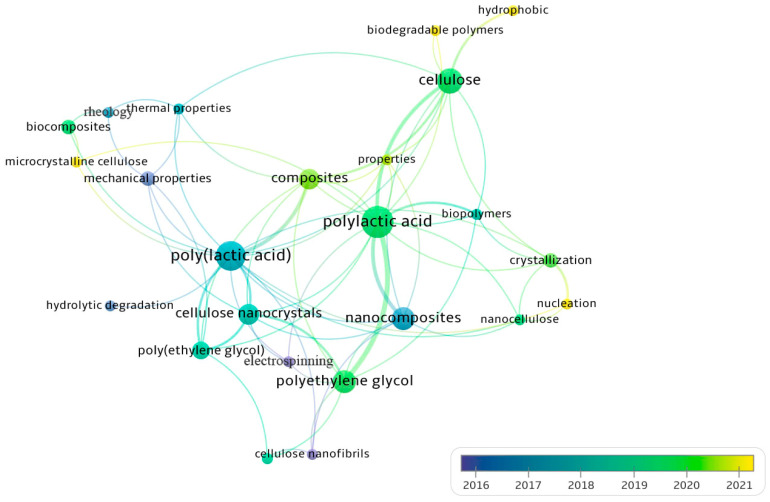
Search 1 keywords networking considering frequency and chronological time in the overlay visualization mode, according to Scopus database.

**Figure 3:**
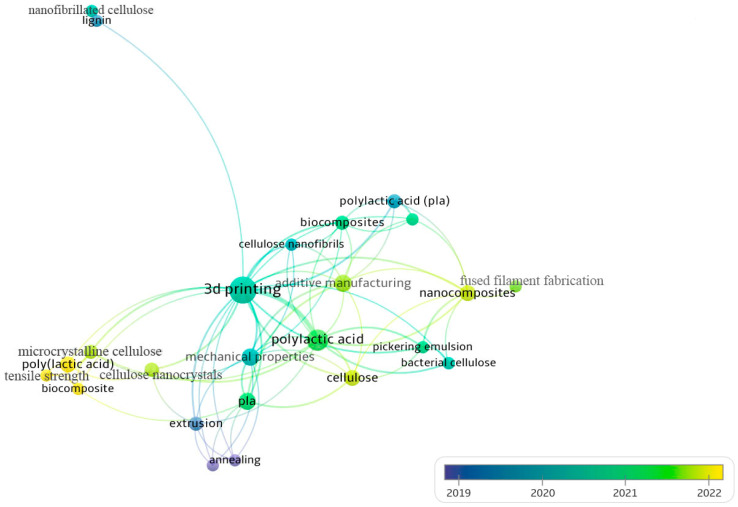
Search 2 keywords networking considering frequency and chronological time in the overlay visualization mode, according to Scopus database.

Keyword networking was realized using VOSviewer software (version 1.6.16) considering the frequency and chronological time (Figure 2 and Figure 3) with a minimum of two keyword occurrences, according to the Scopus database. From Search 1, the most incident clusters are “polylactic acid”, “cellulose”, “polyethylene glycol”, “nanocomposites”, “composites”, and “cellulose nanocrystals”. But the most recent clusters (yellow to green colors) are “microcrystalline cellulose”, “biodegradable polymers”, “hydrophobic”, “properties”, “nucleation”, “composites”, “biocomposites”, “crystallization”, and “nanocellulose”. This result indicates that nanomaterials, cellulose types, and composites properties have been mostly and recently reported regarding PLA/cellulose composites. From Search 2, “3D printing”, “polylactic acid”, “additive manufacturing”, “mechanical properties”, “cellulose”, and “nanocomposite” are the clusters most cited. In addition, poly(lactic) acid, biocomposites, tensile strength, cellulose, nanocomposites, cellulose nanocrystals, microcrystalline cellulose, fused filament fabrication, and additive manufacturing are the latest clusters. Therefore, concerning PLA/cellulose/PEG composites applied to 3D printing, most of the works reported the use of nanomaterials and discussion about mechanical properties, leading to vast options for future works.

#### 6.2.2. Effect of PEG on the Properties of PLA/Cellulose Composites

**Figure 4:**
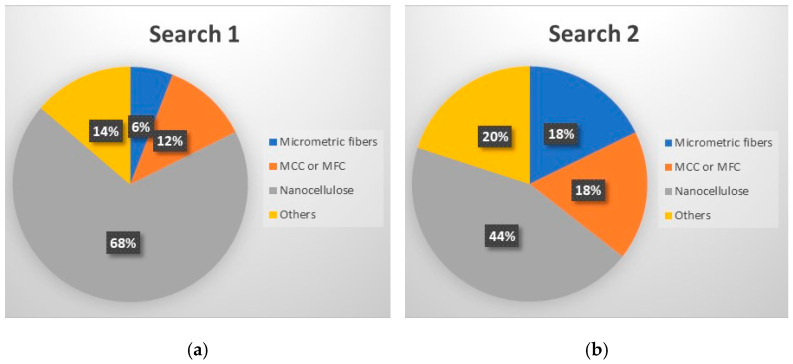
Distributions of publications considering cellulose scale: (**a**) results from Search 1 and (**b**) results from Search 2.

Among the 51 research papers published in the literature using the keywords specified in Search 1 ((“PLA” OR “polylactic acid” OR “poly(lactic acid)” OR “poly(lactide)” AND “cellulose” AND “PEG”), as detailed in Table 4 and Figure 4a, nearly 70% employed nanocellulose (NCC, CNF, or CNS) as a filler. The remaining publications adopted alternative materials, including micrometric fibers (three publications) [43,55,70], microcrystalline cellulose (MCC or MFC) (six publications) [10,17,50,83,84,85], or presented distinct combinations of PLA + cellulose + PEG. These combinations include blends [86,87], sandwich-structured PVA-based PEG–PLA + neat PLA composite films prepared to enhance the hydrophobicity of the PVA + NCC/CNF nanocomposite films [88,89], cellulose film surface treated with PEG and recovered with PLA or HDPE film [90], as well as the use of cellulose derivatives [91,92].

Publications with cellulose fibers (or micrometric fibers) reported the use of filler ranging from 15 wt.% to 25 wt.%, while the amount of PEG reported in these papers was between 0.5% to 40%. Moscoso-Sánchez et al. [70] used 15 wt.% of henequen fiber and low-molecular-weight PEG (20% and 40% of PEG400). The PEG was impregnated in the fiber surface during the steam explosion treatment. The low-molecular-weight PEG was used in this case to improve the fiber–matrix compatibility. As a result, an improvement in the tensile strength and tensile modulus was observed and related to good adhesion. In addition, the PEG impregnated into the fiber reduced the Tg from 59 °C to 52 °C and plasticized the PLA matrix. Two other publications [43,55] reported the use of higher molecular weight PEG (3350 g/mol and 2000 g/mol), respectively. Xie et al. [43] changed the hydrophilicity of PLA/ramie fiber biocomposites to attract more water attack by introducing water-soluble PEG (0.5, 10, and 15 wt.%) and reported that PEG significantly enhanced the surface erosion process and thus facilitated the degradation rate. Addition of 15 wt.% of PEG led to a complete degradation of PLA biocomposites within 50 days. Ding et al. (2016) [55] used PEG2000 as a lubricant in the development of PLA/PEG, PLA/NBSK/PEG, and PLA/MDF/PEG composite foams using injection molding. In this study, the authors did not assess the impact of the additive, as all the samples under investigation included PEG.

The use of MCC was reported by Jirum and Baimark [84], Moreno et al. [85], and Bhiogade and Kannan [83], while the use of MFC was reported by Sirisinha et al. [50] and Molinari et al. [10]. Wang et al. [17] reported the use of micro/nanocellulose (MNC), a mixture of microcellulose fibers and nanocellulose fibers, obtained from a bleached softwood pulpboard from a mechanical treatment and a surface modification using a pretreatment with PEG400 and a silane coupling agent (KH-550). In general, the amount of filler in all these papers ranging from 1 to 50 wt.% and the addition of MCC or MFC provided an improvement in the thermal stability and in the tensile modulus of PLA composites with a consequent decrease in the elongation at break. The crystallinity also decreases with the increase in MCC or MFC amount. 

Wang et al. [17] also noted that incorporating MNC led to a reduction in the melt flow rate of the composites. Notably, a composition containing 30 wt.% of MNC yielded equivalent mechanical properties to those of pure PLA.

PEG was added in these papers in the range from 5 to 20%, and the molecular weights were 400 g/mol [10,85], 600 g/mol [83], 4000 g/mol [50], and 6000 g/mol [17]. Jirum and Baimark [84] was an exception; the authors did not use PEG as an additive but as a blend between PLLA and PEG (flexible poly(L-lactide)-b-polyethylene glycol-b-poly (Llactide)-PLLA-PEG-PLLA). The addition of lower molecular weight PEG (400 and 600 g/mol) decreased the elastic modulus and enhanced the ductility of the matrix, favoring plastic deformation [10], decreasing the crystallinity [85], and reducing the thermal stability [83]. On the other hand, according to Sirisinha et al. [50], the addition of high-molecular-weight PEG (PEG4000) in PLA/MFCs composite not only helped feeding and dispersion of the fibers but also induced cold crystallization of the PLA matrix. Wang et al. [17] reported that the use of PEG (6000 g/mol) increased the melt flow rate of PLA/MNC composites to meet 3D printing demands but decreased the tensile and flexural strength with the increase in the PEG content and increased the elongation at break, only for 5% of PEG content. The authors also reported that the best composition was 30 wt.% of MNC, modified with KH-550, 5% of PEG6000, and 65 wt.% of PLA. 

Most of the research papers therefore used nanocellulose as a reinforcement, both NCC and CNF. Only one publication reported the use of cellulose nanosphere (CNS) [102]. With the use of cellulose at a nanoscale, as already widely reported in the literature, the amount of reinforcement is lower, varying between 0.1 and 10 wt.%. PEG was used in these papers both as a compatibilizing agent to improve the adhesion between cellulose and PLA, being grafted onto the cellulose chain (cellulose-g-PEG), and as plasticizer to increase chain mobility of PLA. The molecular weight of the PEG used ranged from 400 g/mol to 6000 g/mol, apart from the papers of Raisipour-Shirazi et al. [106], Safdari et al. [109], and Aouay et al. [111], who used PEG with higher molecular weight values, such as 180,000 g/mol, 20,000 g/mol, and 35,000 g/mol, respectively. In general, authors who used PEG as a coupling agent reported the use of PEG with molecular weight of around 600 to 750 g/mol.

The use of PEG as a coupling agent to change the surface properties of cellulose and increase the compatibility with PLA was reported by Macke et al. [94], Yu et al. [102], Zhang et al. [81], Gois et al. [105], Geng et al. [19], Raisipour-Shirazi et al. [106], Aouat et al. [107], Fujisawa et al. [80,115], and Li et al. [116]. Macke et al. [94] studied the effects of polymer grafting density and molecular weight of PEG on the properties of PLA composites and reported that NCCs grafted with lower molecular weight PEG (<2000 g/mol) had significant embrittlement, while PEG with higher molecular weight (10,000 g/mol) avoided the embrittlement of the PLA matrix.

Srisawat et al. [93] compared the effect of two commonly used plasticizers, PEG and poly (butylene adipate) PBA, and observed that PEG (4000 g/mol) increased the ductility of composites, while composites containing PBA plasticizer exhibited higher modulus, strength, and heat resistance.

Eicher et al. [96] reported the use of biobased materials to improve the mechanical properties of PLA composites. NCC (0.25–1%) and dried distillers’ grains with solubles (DDGS) (10%) were used as reinforcing agent, PEG (400 g/mol) as plasticizer, and maleic anhydride (MA) as a coupling agent. From the results, the authors observed that NCC and PEG400 improved the mechanical properties (Young’s modulus and ultimate tensile strength) of PLA, whereas MA aids in the dispersion of NCC.

In general, higher plasticizer molecular weights are associated with increased resistance to migration, prolonged utility lifespan, and reduced volatilization. Nevertheless, based on the results published in the literature for PLA/cellulose composites and nanocomposites, PEG can act as a plasticizer, its most common use. Additionally, PEG serves as a compatibilizer between hydrophilic cellulose and hydrophobic PLA. Thus, the effect of PEG addition can vary according to its molecular weight, the added content, as well as the specific type and scale of cellulose employed as a filler or reinforcement.

The primary manufacturing processes reported in the publications arising from Search 1, for composites reinforced with fibers and MCC or MFC, were the combination of mixing processes such as melting blending using an internal mixer or twin-screw extruder, with molding processes such as solvent casting, compression, extrusion, and injection (Table 4). For nanocomposites, solvent casting and solvent casting followed by compression molding were the processes most reported. From all publications, only the study reported by Wang et al. [17] performed PLA/cellulose composite filaments production and 3D printing.

#### 6.2.3. PLA/Cellulose Composites for 3D Printing Filament Production

In the systematic examination of the literature using the terms specified in Search 2 ((PLA” OR “polylactic acid” OR “poly (lactic acid)” OR “poly(lactide)” AND “cellulose” AND “3D printing”), a total of 45 published papers were identified (as outlined in Table 5 and represented in Figure 4b). Those works, in various capacities, discuss the application of PLA and cellulose in 3D printing, predominantly employing the fused filament deposition (FFD) method. However, within this set of works, nine manuscripts diverge from the focus on PLA/cellulose composites. Instead, they explore blends (such as PLA/PCL) [119], the utilization of cellulose derivatives (like hydroxypropyl-methylcellulose) [120], or the integration of hemicellulose (such as galactoglucomannan (GGM)) as a filler for PLA [121].

Most published studies regarding cellulose-reinforced PLA composites center around the use of natural reinforcement at the nanometer scale. Drawing from the bibliometric investigation conducted, among the papers stemming from Search 2, approximately 44% feature nanocellulose (NCC or CNF), 18% involve MCC or MFC, and another 18% incorporate cellulose fibers sourced from various natural origins (as depicted in Figure 4b). Interestingly, the distribution of publications, when considering cellulose scale, exhibits a more uniform pattern in Search 2 compared to the variations observed in Search 1 (as illustrated in Figure 4).

Within the subset of eight publications that explored cellulose at the micrometric scale (cellulose fibers), the content of fibers integrated into the PLA matrix spanned from 3 to 30 wt.%. Notably, merely two of these publications documented the implementation of surface modifications to the fibers. Jiang et al. [125] documented the chemical modification of wood with 3-aminopropyltriethoxysilane (APTES). In a separate study, Ma et al. [122] conducted a comparative analysis involving three different pretreatment methods for reed (NaOH, p-TsOH (p-toluenesulfonic acid, and H_2_SO_4_-SE-steam explosion). Their evaluation encompassed both the potential for ethanol production and the enhancement of economic viability in the reed biorefinery process. The outcomes of this analysis led to the identification of three specific pretreatment processes: RES (enzyme hydrolysis-processing residue treated with NaOH), REP (enzyme hydrolysis-processing residue treated with p-TsOH), and RED (enzyme hydrolysis-processing residue treated with H_2_SO_4_-SE). Subsequently, these treated residues were harnessed as the reinforcing phase for the material utilized in 3D printing.

It is worth mentioning that two other publications, related to the keywords of Search 2 and using cellulose fibers as a filler, were identified in the literature, although they did not appear in the searches carried out in the databases used, probably because they did not contain the exact keywords used in this search. In these two publications, the authors also used surface treatment of the cellulose fiber, such as treatment with tetraethyl orthosilicate (TEOS) to increase the compatibility between PLA and the fiber [20] and chemical treatments of alkalinization and silanization, reported by [16], for improving adhesion and removing impurities.

The application of PEG was documented by Ma et al. [122], who incorporated 3% of PEG600 and 4% of KH550 as coupling agents in their work. However, the authors did not provide a report on the impact of PEG addition on the material’s properties. Additionally, Aumnate et al. [20] explored the integration of PEG (with molecular weights of 4000 and 6000 g/mol) to enhance the flowability and processability of biocomposite filaments composed of polylactic acid filled with 10 wt.% of kenaf cellulose fibers. Their findings indicated that the incorporation of kenaf fibers led to an increase in melt viscosity, while the introduction of plasticizers like PEG resulted in viscosity reduction. Notably, higher molecular weight PEG (PEG6000) imparted more hydrophobic properties. Furthermore, the addition of PEG improved the dispersion of kenaf cellulose fibers within the PLA matrix. This addition also led to a decrease in glass transition temperature and complex viscosity, along with a reduction in storage moduli across all frequencies. Interestingly, the higher molecular weight PEG (PEG6000) improved the tensile strength of biocomposites.

In terms of employing MCC or MFC, the database search yielded eight publications. Within this set, seven studies employed MCC, with only one publication opting for MFC. Winter et al. [129] presented the advantages of MFC containing residual lignin and hemicellulose compared to MFC from bleached pulp in the filament production of MFC/poly(lactic acid) for 3D-printing applications. The authors reported a considerable reduction in MFC agglomeration and an improvement in the printability and toughness of printed objects.

The amount of MCC and MFC reported in these works varied from 1 wt.% to 50 wt.%, and the use of a superficial modification of cellulose was reported by Murphy et al. [5] and by Winter et al. [129]. Murphy et al. [5] reported that cellulose was surface-modified using a titanate coupling agent and that the addition of modified cellulose to the PLA matrix increased the storage modulus of biocomposites and increased the mobility of the PLA chains. Winter et al. [129] carried out different pre-chemical treatments on the cellulose surface and used as a filler MFC with different contents of lignin and hemicellulose. Other strategies to improve cellulose/PLA adhesion were also reported by Wang et al. [17] who used a silane coupling agent (KH550) and by Ye et al. [48] who studied the addition of maleic anhydride (MA) copolymer on the PLA/polycaprolactone (PCL)/MCC composites.

From Search 2, 20 documents related to the use of nanocellulose as a filler to PLA matrix. Besides NCC and CNF, the use of carbonized cellulose nanofibers [131] and TEMPO-oxidized bacterial cellulose [142] are also reported. To improve the compatibility of the nanocellulose with a hydrophobic matrix of PLA, many works report the use of some modification in the cellulose, such as graphitization of CNF with PLA [25,77,130], polymerized lignin surface-modified CNF [134], and the use of TEMPO [137,142]. The most used nanocellulose isolation methods were acid and enzymatic hydrolysis for the isolation of NCC and mechanical processing for isolation of CNF, with contents varying between 0.1 and 5 wt.%, apart from the papers [25,132] which used a maximum of 20 wt.% and 40 wt.% of filler, respectively.

The effect of NCC or CNF addition on the PLA matrix, however, depends on different factors such as amount of filler added and the use of a coupling agent or some cellulose surface modification. According to Dong et al. [130], the incorporation of PLA-g-CNFs improved storage modulus of the composite filaments in both the low-temperature glassy state and high temperature rubbery state. Gregor-Svetec et al. [134], for example, reported that the addition of NFC slightly reduced tensile strength, stretchability, and ability to absorb energy of the filaments, while the initial modulus slightly improved.

Among the papers resulting from Search 2, not all reported studies considered the 3D printing process; some publications reported only filament production, using in most cases a twin-screw extruder (Table 5). In some research papers, the melt mixture between the matrix and the reinforcement was carried out prior to extrusion, while in other publications, extrusion in a twin-screw extruder served both as a melt mixing step to obtain the composite/nanocomposite as well as to produce the filament.

Table 6 summarizes some of the main parameters used for the 3D printing process reported in the research papers resulting from Search 2.

As in Search 1, some papers resulting from Search 2 did not necessarily report the production of PLA/cellulose composites or nanocomposites but rather the formation of blends and/or the use of cellulose derivatives. Pis et al. [143], for example, did not produce the PLA/cellulose filament but studied the properties of different commercial filaments such as transparent PLA (referred to as PLA), PLA–wood (referred to as pine), PLA–cork (referred to as cork), PLA–bamboo (referred to as Bambus), while Jiang et al. [120] prepared hydroxypropyl methylcellulose (HPMC)/PLA composite filaments to produce parts via fused deposition modeling (FDM).

Finally, from Search 3 (“PLA” OR “polylactic acid” OR “poly(lactic acid)” OR “poly(lactide)” AND “cellulose” AND “PEG” AND “3D printing”), only two research papers were identified. These two works are from the same authors, in which they discussed the use of PLA with NCC concerning the printability of the composite plasticized with PEG [17] and also focusing on the kinetic study of the samples [52]. Moreover, both articles appeared in Searches 1 and 2 and have been cited in the previous sections.

## 7. Prospects

In the realm of PLA/cellulose (nano)composites and 3D printing, there are numerous promising avenues for future exploration and research.

Firstly, the potential of cellulose in these composites can be further unlocked by varying its scale, content, and sourcing from different natural origins. By tailoring the type and source of cellulose, researchers can fine-tune the composite properties, adapting them to specific applications. Furthermore, there is a critical need to delve deeper into understanding how different molecular weights of polyethylene glycol (PEG) influence PLA/cellulose (nano)composites. This investigation is essential for optimizing composite properties and ensuring they meet desired performance criteria.

In the context of 3D printing, one intriguing prospect is addressing the challenges associated with multiple extrusion cycles, a common practice for achieving better composite uniformity. Given PLA’s sensitivity to temperature and potential degradation, exploring the effects of multiple extrusion cycles is vital to preserving material integrity. The rapidly evolving landscape of 3D printing, as an emerging manufacturing technology, necessitates increased attention and diversified research. This entails exploring innovative approaches, materials, and processes, particularly within the realm of composites.

Environmental considerations are becoming increasingly important. Future research should encompass comprehensive assessments of the environmental impact of PLA/cellulose composite filaments and the final printed products. Sustainability, carbon footprint, and eco-friendliness throughout their lifecycle should be thoroughly evaluated. Understanding the end-of-life implications of PLA/cellulose (nano)composites is equally crucial. This includes studying the disposal and recyclability aspects to ensure these materials align with sustainable practices and do not contribute to environmental harm.

Lastly, evaluating the residue generated during filament production and 3D printing processes is vital. Effective waste management strategies should be developed to minimize environmental impact and maximize resource utilization.

In summary, the prospects for future research in the field of PLA/cellulose (nano)composites and 3D printing are expansive, encompassing various opportunities from material composition enhancement to sustainable practices and environmental impact assessment. Addressing these prospects will drive progress in the field, optimizing composite performance and promoting sustainability in 3D printing. Future research should also focus on the environmental impact of the PLA/cellulose composite filaments and final printed products, as well as their end of life. Also, the residue generated throughout the filament and printing process should be evaluated.

## 8. Applications

The versatility of PLA/cellulose (nano)composites, particularly when enhanced with additives like polyethylene glycol (PEG), extends to a wide array of industries and uses. In the realm of packaging, these composites offer an eco-friendly alternative. They can be employed for biodegradable packaging solutions, including food packaging [149,150,151], single-use items [10], and disposable cutlery [152], contributing to the reduction in environmental impact.

In the biomedical field, the biocompatibility of these (nano)composites makes them suitable for creating biodegradable medical devices. They have applications in biodegradable implants, drug delivery systems, and tissue scaffolds for regenerative medicine [20,153,154].

In the realm of 3D printing, neat PLA is already widely employed for producing biodegradable and environmentally friendly 3D-printed objects. This includes a wide range of items from prototypes to functional parts and artistic creations. However, when combined with cellulose as a reinforcement, the literature predominantly focuses on material characterization and filament production, with limited reporting on specific practical applications. Nevertheless, the PLA/cellulose (nano)composite shows significant promise for 3D printing applications, especially when incorporating additives like PEG. These additives enhance the composite’s rheological, mechanical, and thermal properties, making it an attractive candidate for a wide range of 3D printing applications [5,6,17,24,42,44,47,48,51,53,77,120,122,125,126,131,136,142,143].

In summary, PLA/cellulose (nano)composites have some other less common yet promising applications including textiles, automotive, agriculture, consumer goods, and construction. These applications offer sustainable solutions and align with environmental responsibility. Ongoing research is expected to reveal more innovative uses in the future.

## 9. Conclusions

This review covered the main concerns on PLA/cellulose/PEG composites. Firstly, an overview of each material was given, emphasizing its recent trends. Then, a systematic review was provided according to the most current databases in the field (Scopus and Web of Science), in which three searches were highlighted and discussed over the work.

Cellulose, as a valuable compound from natural sources, has been widely applied in the PLA composites field with articles published in the past 45 years (848 documents), while the use of PEG (Search 1) and the composite’s application in 3D printing (Search 2) are recent subjects. Regarding the 3D printing topic, Search 2 presented 39 documents, with an increasing tendency in the last 6 years, emphasizing that this subject should be explored more in future works, focusing on improving the PLA/cellulose filaments’ performance in printing. Also, Search 3 showed that the use of PEG to improve the 3D printability of PLA/cellulose composite represents a significant gap in the literature as well as the use of other plasticizer/coupling agents.

The discussion of the articles resulting from Searches 1 and 2 provided significant information about the scales/content of cellulose that have been used, the principal properties of the composites, and possibilities for improvement by using a plasticizer with different molecular weight. Also, printing parameters and performance were widely reported. Therefore, this work not only provided valuable information to guide future studies but also emphasized 3D printing as an innovative feature for PLA reinforced with natural filler composites, showing that there is much more than the commonly used PLA filament.

## Figures and Tables

**Table 1:** Comparison between ABS, PLA, PETG, and PP properties considering 3D printing.

Properties	ABS	PLA	PETG	PP
Tensile strength (MPa)	40	65	53	32
Stiffness	5/10	7.5/10	5/10	4/10
Durability	8/10	4/10	8/10	9/10
Maximum service temperature (°C)	98 °C	52 °C	73 °C	100 °C
Coefficient of thermal expansion (µm/m °C)	90	68	60	150
Density (g/cm^3^)	1.04	1.24	1.23	0.9
Price (dollar/kg)	10–40	10–40	20–60	60–120
Printability	8–10	9–10	9–10	4–10

Source: Simplify3D Software Library (version 5.1.2).

**Table 2:** Systematic search in the Scopus and Web of Science databases.

Search	Terms	Scopus	Web of Science	Total Number of Documents	Years of Publication
0	(*“PLA” OR “polylactic acid” OR “poly(lactic acid)” OR “poly(lactide)” AND cellulose AND composite*)	848	498	1346	1978–2023
1	(*“PLA” OR “polylactic acid” OR “poly(lactic acid)” OR “poly(lactide)” AND “cellulose” AND “PEG”*)	45	06	51	2006–2023
2	(*“PLA” OR “polylactic acid” OR “poly(lactic acid)” OR “poly(lactide)” AND “cellulose” AND “3D printing”*)	39	06	45	2017–2023
3	(*“PLA” OR “polylactic acid” OR “poly(lactic acid)” OR “poly(lactide)” AND “cellulose” AND “PEG” AND “3D printing”*)	02	0	02	2017–2020

**Table 3:** The five most cited documents, according to Scopus database.

Reference	Country	Scopus	Citations ^1^	Evolution of Citations
Search 1 ^2^				
[57]	Norway	*Comp. Sci. Tech.*	680	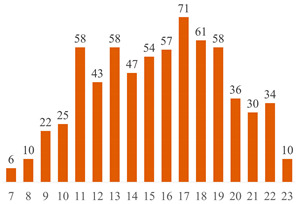
[79]	China	*Bioresources*	192	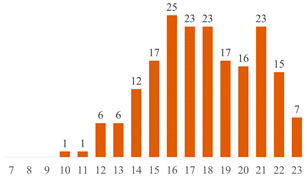
[80]	Japan	*Biomacrom.*	162	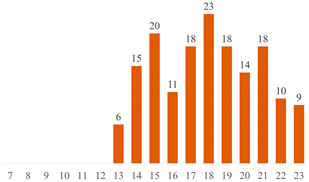
[81]	China and USA	*Mat. Sci. Eng. C*	125	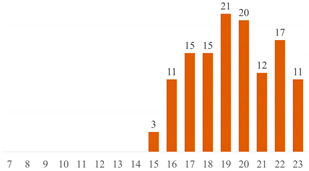
[17]	China	*Ind. Crops and Products*	70	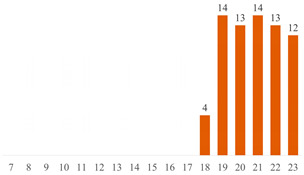
Search 2 ^3^				
[5]	Ireland	*Pol. Comp.*	143	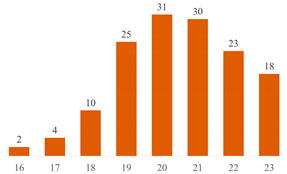
[13]	China	*Pol. Adv. Tech.*	90	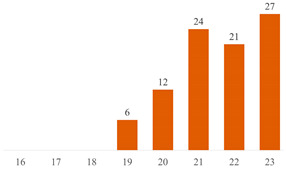
[82]	Romania	*Nanomaterials*	73	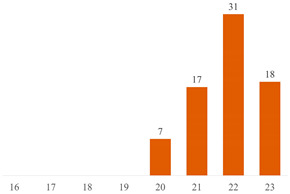
[17]	China	*Ind. Crops and Products*	70	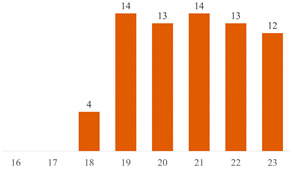
[47]	Canada and USA	*Materials Today Sustainability*	62	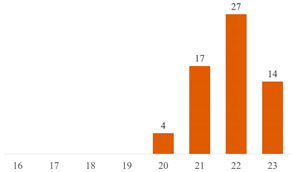

^1^ until 13 July 2023, ^2^ 2007–2023, ^3^ 2016–2023.

**Table 4:** Technical details regarding the documents obtained from Search 1.

Reference	PLA	Filler (wt.%)	Modification	Plasticizer (%)	Manufacturing
Fibers					
[70]	2003D	Henequen (15)	PEG impregnated by steam explosion	PEG400 (20 and 40)	Batch mixer and compression molding
[43]	4032D	Ramie (20)	-	PEG3350 (0.5, 10, and 15)	Corotating twin-screw extrusion and injection molding
[55]	4032D	Softwood kraft or black spruce fiberboard (25)	-	PEG2000 (0.5)	Twin-screw extrusion and injection molding
MCC					
[17]	PLA-M_w_ 100.000	MCC and NCC (10, 30, and 50)	Silane-coupling agent (KH-550)	PEG6000 (5, 10, and 20)	Casting and extrusion
[83]	3251	MCC and NCC (1, 3, and 5)	-	PEG600 (10 and 15)	Casting
[10]	2003D	MFC (2)	-	PEG400 (5, 10, and 15) and OLA ^1^	Ultra turrax
[85]	7000D	MCC from pineapple leaf fibers (2, 4, 6, 8, and 15)	-	PEG400 (10) and TBA ^2^ as surfactant	Casting
[84]	PLLA ^3^ and PLLA-PEG-PLLA ^4^	MCC (5, 10, and 20)	-	-	Batch mixer and compression molding
[50]	2003D	MFC (3 and 5)	-	PEG4000 (5)	Internal mixer
NCC/NCF					
[93]	2003D	NCF from rubberwood sawdust (1)	-	PEG4000 (15) and PBA5000	Internal mixer and compression molding
[94]	4060D	TEMPO NCF (1, 2, 5, and 10)	Grafted with PEG	PEG550	Solvent casting
[95]	4032D	NCF from eucalyptus bleached kraft pulp (1.3 and 5)	-	PEG4000 and PEG1500 (7.5)	Mixed in a torque rheometer and injection molding
[96]	2003D	NCC (0.25, 0.1, and 1) and DDGS ^5^ (10)	Grafting with maleic anhydride (0.25)	PEG400 (2)	Mechanical grinder, twin-screw extrusion and injection molding
[97]	4032D	NCF from eucalyptus bleached kraft pulp (1.3 and 5)	-	PEG1500 (7.5)	Mixed in a torque rheometer and compression molding
[98]	3251D	Lignin-NCF from palm waste (2–5)	-	PEG1000 (20)	Twin-screw micro-compounder and extrusion
[83]	3251	NCC and MCC (1.3 and 5)	-	PEG600 (10 and 15)	Solution casting
[99]	PLA-M_w_ 100.000	NCC (2)	-	PEG100, PEG4000, and PEG6000 (4)	Mixed in NCC suspension, scraped, and dried (electric heating board)
[52]	4032D	NCF enzymatic from MCC (1, 2.5, and 5)	-	PEG600 (4)	Mixing and single-screw extrusion
[100]	4032D	NCF enzymatic (5)	-	PEG2000 (10 and 20)	Mixing at aqueous suspension and twin-screw compounder
[101]	PLA-M_w_ 150.000 D	NCC (1, 2, 3, and 5)	Modifier with TEMPO	PEG-NH_2_ (adsorption on NCC surface)	Mixing and casting
[102]	PLA-M_n_ 1.0 × 10^5^ g/mol	NCS (10)	Grafting with PEG	PEG6000, PEG4000, and PEG2000	Solvent casting
[81]	2002D	NCC (1, 5, and 10)	Grafting with PEG	PEG5000	Electrospun
[103]	2003D	NCC (1, 2, 4, 6, 8, and 10)	-	PEG6000 (4, 6, 8, 10, 12, and 20)	Melt blended and solvent casting
[104]	2003D	NCC (1, 2, 4, and 6)	-	PEG6000 (10, 12, 14, and 16)	Solvent mixing and melt blended
[105]	3251D	NCC (3)	Modified with surfactant	PEG300, PEG1000, PEG monooleate and PL44 ^6^	Solvent casting
[19]	4032D	NCF (0.1)	Modified with TEMPO and grafted with PEG	PEG-NH_2_ and methoxy-PEG (0.05)	Casting and compressing molding
[106]	2003D	NCF (10)	Acetylation and grafting with PEG	PEG180000	Solvent casting
[107]	6202D	MCC and NCW commercial (1 and 3)	Grafting with maleic anhydride	PEG1500 (10)	Twin-screw extrusion and spinning
[108]	PLA-M_n_ 64.166 g/mol	Bacterial NCC (1, 2.5, 5, and 10)	PLA/PEG copolymer	PEG-M_n_ 62.07 g/mol	Solvent casting and particulate leaching methods
[109]	3251D	NCF (2 and 5)	-	PEG20000 (4)	Solution casting and direct melt mixing
[18]	4032D	NCC (5, 10, and 20)	PEG as surfactant	PEGME ^7^ -M_n_ = 2000 g/mol (10)	Twin-screw extrusion and injection molding
[110]	PLA from Nature Works (D-isomer 2%)	Bacterial NCW (5)	-	PEG900 (8)	Electrospinning
[79]	PLA-M_w_ 100.000 g/mol	NCF (3)	Grafted with PEG	PEG1000 (2)	Solvent casting
[57]	4031D	NCW from MCC (5)	Grafted with maleic anhydride	PEG1500 (15)	Twin-screw extrusion
[111]	PLA from NaturePlast	NCF, NCF–lignin, NCC, NCC–chitin, and NCC–starch (1)	PEG as a carrier for NCF and NCC	PEG-M_w_ 35.000 g/mol (5)	Casting
[112]	4043D	MFC (5, 10, 15, 20, 25, and 30)	Grafting with PEG	PEG800 (1:2 MFC)	Solvent casting
[113]	3001D	Commercial NCF (0.05, 0.3, 0.6, and 1.3)	Grafting with PEG and capped with a 12-carbon aliphatic chain	PEG600 (5)	Melt spinning
[114]	3001D	NCC and NCF (0.005 and 0.55)	Grafting with PEG	PEG600 (5)	Mechanical mixer and ultrasonication, and twin-screw micro-compounder
[80]	PLLA-M_w_ 94.000 and M_n_ 45.000 g/mol	NCC (0.1, 0.25, 0.5, and 1)	Modified with TEMPO and grafting with PEG	PEG(2182)-NH_2_ (SUNBRIGHT MEPA-20H)	Casting
[115]	PLLA-M_w_ 94.000 and M_n_ 45.000 g/mol	TEMPO NCC/PEG (0.5 and 1)	Modified with TEMPO and grafting with PEG	PEG(23)-NH_2_ and PEG(48)-NH_2_	Casting
[19]	4032D	TEMPO NCF (0.1)	Grafting with PEG	PEG-NH2-M_n_ 750	Solvent casting and compression molding
[116]	4032D	NCC from cotton cellulose (0.1 and 0.5)	Dopamine-induced functionalization	PEG1000 and PEG2000	Solvent casting and compression molding
[117]	PLA from Nature Works^®^	NCC (1) and graphene nanoparticles (15)		PEG4000 (20)	Solvent casting
[118]	2002D	NCC (1, 3, and 5)	Grafting with poly acrylic acid	PEG-M_w_ 10 kDa and OH-terminated)	Twin-screw extrusion
Blends PLA/PEG/cellulose					
[86]	PLL ^8^ 4042D film grade	Cellulose acetate butyrate (10–90)	-	PEG400, PEG1500, and PEG6000 (10, 20, and 30)	Solution casting
[92]	4043D	dialdehyde cellulose from MCC (1:1)	-	PEG6000 (10)	Solvent technique
[91]	PLA	Starch, chitosan, and ethyl cellulose (20 and 30)	-	PEG600 (10, 20, and 25)	Brabender mixer
[87]	2002D	Wood cellulose microfiber (10)	-	PEG600 (2)	Melt blending process and compression molding
[90]	HD5148 (PLA film)	Cellulose fiber (square samples with 120 mm in length)	-	PEG-Brij^®^ 93, O10 and 98 and PEG-M_n_ 1400, 920 and 875 g/mol (2.5)	Compression molding
[88]	2003D	NCC and NCF	-	PEG-M_w_ 1900–2200	Solution casting and heat treatment
[89]	2003D	NCC and NCF	-	PEG-M_w_ 1900–2200	Double-sized solution casting and heat treatment

^1^ oligomeric lactic acid, ^2^ tert-butanol, ^3^ poly(L-lactic acid), ^4^ poly(L-lactide)-b-polyethyleneglycol-b-poly(L-lactide), ^5^ dried distillers’ grains with solubles, ^6^ pluronic^®^ L44, ^7^ Poly(ethylene glycol) monomethyl ether, ^8^ Poly(L-lactide).

**Table 5:** Technical details regarding the documents obtained from Search 2.

Reference	PLA	Filler (wt.%)	Modification	Plasticizer (%)	Manufacturing
Fibers					
[13]	4032D	Cellulose from sugarcane bagasse (3, 6, 9, 12, and 15)	-	-	Twin-screw extrusion and 3D printing
[122]	4032D	EPR ^1^ (20)	NaOH, p-TsOH ^2^, and H2SO4-SE ^3^ pretreatments	PEG600 (3) and KH550 ^4^ (4)	Twin-screw extrusion, injection molding and 3D printing
[123]	PLA/PHB ^5^ (3:7, 1:1 and 7:3)	Cellulose fibers (1, 5, and 10)	-	Polyoxylethylene 400 (4)	Single-screw extrusion and 3D printing
[124]	PLA2003D/PBAT (1:10)	Cellulose wood fibers (0.2)	-	-	Twin-screw extrusion
[45]	4032D	Bagasse fibers—80, 120, and 200 mesh sieved (10, 15, 25, 40, and 50)	Grafted with glycidyl methacrylate (GMA)	-	Twin-screw extrusion, then filaments were cut into pellets and 3D printed
[125]	PLA	Wood powder (85)	Modified with APTES ^6^	-	Extrusion and 3D printing
[126]	2003D	Lyocell fibers (30)	-	-	Twin-screw extrusion and 3D printing
[42]	2003D	Regenerated cellulose fibers (10, 20, and 30)	-	-	Twin-screw extrusion and 3D printing
MCC					
[5]	3001D	MCC (1, 3, and 5)	Modified with titanate coupling agent	-	Casting, extrusion, and 3D printing
[127]	PLA from Nature3D (Japan)	MCC (3)	Gamma-ray irradiation	-	Single-screw extrusion and 3D printing
[47]	4043D/PLA recycled (30%)	MCC (5)	Modified with epoxy-based chain extender	-	Manual mixing, twin-screw extrusion, and 3D printing
[17]	PLA-M_w_ 100.000	MCC and NCC (10, 30, and 50)	Modified with KH550	PEG6000 (5, 10, and 20)	Casting, twin-screw extrusion, and 3D printing
[128]	PLA or cellulose acetate propionate	MCC (20)	Cellulose acetate propionate with plasticizer	Phthalate free plasticizer	Twin-screw extrusion, injection, and 3D printing (pellets)
[48]	4032D/PCL ^7^ (20%)	MCC (5, 10, 15, and 20)	Grafted with maleic anhydride (3%)	-	Melt blending, twin-screw extrusion
[129]	4043D	MFC from beech wood (1)	Cellulose fibrillated, partially delignified/fibrillated and conventional fibrillated from bleached softwood pulp	-	Twin-screw extrusion (twice) and 3D printing
[6]	4043D	MCC (6, 9, 12, and 18)	-	-	Twin-screw extrusion, single-screw extrusion, and 3D printing
NCC/NCF					
[130]	4032D	Commercial NCF (1, 3, and 5)	Grafted with L-lactide monomers	-	Melt compounding and extrusion
[77]	4032D	Commercial NCF (1 and 3)	Grafted with L-lactide monomers	-	Extrusion and 3D printing
[131]	4032D	Carbonized NCF	-	-	Single-screw extrusion and 3D printing
[132]	4043D	NCF (10-4)	-	-	Plunger-type batch extrusion and compression molding or 3D printing
[133]	cPLA1001/PBS ^8^	NCF from saw dust (1, 3, and 5)	Modified with canola oil	-	Twin-screw extrusion, injection molding, and 3D printing
[134]	2003D	NCF–lignin (1, 3, and 5)	Modified by electrostatic adsorption of lignin	-	Melt compounding and twin-screw extrusion
[135]	3251D	Enzymatic NCF (1 and 3)	Enzymatic pretreatment	-	Melt compounding, extrusion, and 3D printing
[25]	2003D	Commercial NCF (1, 2, 3, 10, and 20)	NCF grafted by a solvothermal reaction	-	Melt compounding, extrusion, and 3D printing
[82]	PLA4043D/PHB (75:25)	NCF from plum seed shells (1)	Dicumyl peroxide as a cross-linking agent (1%)	-	Melt blending and compression molding or twin-screw extrusion and 3D printing
[53]	3051D	NCF from sisal fibers (1, 3, and 5)	-	-	Casting, extrusion, and 3D printing or compression molding
[52]	4032D	NCF (1, 2.5, and 5)	Enzymatic pretreatment and high-pressure micro-fluidization	PEG600 (4)	Single-screw extrusion
[136]	4032D	Enzymatic NCF from MCC (1, 2.5, and 5)	NCF was isolated by enzymatic hydrolysis	PEG600 (4)	Extrusion and 3D printing
[24]	2002D	Commercial NCF (0.75, 1 and 2)	-	-	Single-screw extruder and 3D printing
[137]	PLA/PCL (80:20)	TEMPO NCF (1.5)	Modified with TEMPO	-	Pickering emulsion, single-screw extrusion, and 3D printing
[138]	PLA from Nature Works	NCC from *Ficus thonningii* (1, 3, and 5)	Dissolved in dichloromethane	-	Single-screw extrusion and 3D printing
[51]	6202D	NCC from eucalyptus (0.1, 0.5, and 1)	-	PEG, triacetin, and Joncryl ADR-436-C ^9^	Single-screw extrusion and 3D printing
[139]	3052D	NCF (0.5, 1, 2, and 3)	Cu_2_O as antibacterial (0.5)	-	Extrusion and 3D printing
[140]	4043D	NCF (5 and 10)	-	-	Micro-compounder, extrusion and 3D printing
[141]	PLA-M_w_ 18.500	NCC (20, 40, 60, and 80)	Modified with 2-aminoethyl methacrylate	PEGDA ^10^-M_w_ 700 or GelMa ^11^ (20)	Cryogelation and 3D printing
[142]	4043D	TEMPO bacterial cellulose (1, 1.5, 2, and 2.5)	TEMPO bacterial cellulose via Pickering emulsion approach	-	Single-screw extrusion and 3D printing
Blends PLA/PEG/cellulose					
[120]	4032D				
[119]	PLA4032D/PCL				
[143]	PLA commercial filament	Wood, bamboo, and cork (40)	-	-	3D printing
[121]	4043D				
[144]	L-PLA (1, 1.5 and 2%)	Bacterial cellulose membrane	-	-	PLA was printed onto the cellulose membrane (3D layer printing)
[145]	PLA3D700/PHB				
[146]	4032D (50%)	Carbonyl iron powder (12–14) and reduced graphene oxide (3–6)	Ethyl cellulose as the backbone and epoxy resin as the bonding agent (15–17% each)	-	Micro-emulsion method, ball-milling, extrusion, and 3D printing
[147]	PLA	Cellulose (20 and 40)	-	-	Optimization of 3D printing parameters
[148]	PLA filament	Gelatin–carboxymethylcellulose–alginate	Prepared as bioink	-	3D printing (PLA as a negative mold)

^1^ enzyme hydrolysis-processing residue, ^2^ (p-toluenesulfonic acid), ^3^ steam explosion, ^4^ silane coupling agent, ^5^ poly (3-hydroxybutyrate) ^6^ 3-aminopropyltriethoxysilane, ^7^ poly (ɛ-caprolactone), ^8^ polybutylene succinate, ^9^ a food-safe styrene-acrylic multifunctional chain extender, ^10^ poly(ethylene glycol) dimethacrylate, ^11^ methacrylated gelatin.

**Table 6:** Main parameters used to 3D printing in research papers resulting from Search 2.

Ref.	Nozzle Diameter (mm)	Layer Hight (mm)	Nozzle Temperature (°C)	Bed Temperature (°C)	Infill Density (%)	Raster Angle	Printing Speed (mm/s)
[44]	0.6	0.1	200	50 °C	100		40
[122]	Did not report printing conditions						
[125]	Did not report printing conditions						
[126]	0.75	0.2	210	50	100	0°/90°	30
[42]	0.5	0.1	215	70	100	0°	30
[5]	Did not report printing conditions						
[47]		0.38	200	60 °C	100	45°/45°	60
[17]	0.4	0.2	190	60			50
[48]		0.2	205				50
[6]		0.1	180 and 190	60		45°/45°	
[77]	0.6	0.2	210	60	100		20
[131]	0.4	0.2	220	70	100	+45°/−45°	40
[53]	0.6	0.2	180	60	100		45
[136]	0.4		210		10 and 35		40
[24]	0.4	0.2	215	60			
[51]	0.4		190	50			60
[142]	0.4		220	60	100		75
[120]	0.4	0.2	200	40	100		50
[143]	0.6	0.2	200	50	100	+45°/−45°	45

## Data Availability

Not applicable.
